# Draft genome sequence of *Lactobacillus helveticus* OSU-BDGOAK2 and *Lactobacillus kefiranofaciens* OSU-BDGOA1, kefir grain isolates with potential antibacterial activity

**DOI:** 10.1128/MRA.00304-23

**Published:** 2023-08-01

**Authors:** Brianda D. González-Orozco, Tasha M. Santiago-Rodriguez, Israel García-Cano, Rafael Jiménez-Flores, Valente B. Alvarez

**Affiliations:** 1 Department of Food Science and Technology, The Ohio State University, Columbus, Ohio, USA; 2 Diversigen, Inc., New Brighton, Minnesota, USA; 3 Department of Food Sciences and Technology, National Institute of Medical Sciences and Nutrition Salvador Zubirán, Mexico City, Mexico; University of Rochester School of Medicine and Dentistry, Rochester, New York, USA

**Keywords:** probiotics, kefir, antibacterial activity

## Abstract

We present the draft genome sequence and assembly of *Lactobacillus helveticus* OSU-BDGOAK2 and *Lactobacillus kefiranofaciens* OSU-BDGOA1 isolated from kefir grains that exhibited *in vitro* antibacterial activity against *Escherichia coli* ATCC 25922, *Listeria innocua* ATCC 51742, and *Staphylococcus epidermidis* ATCC 1222. Genome analysis of both strains revealed gene clusters encoding bacteriocins.

## ANNOUNCEMENT

Milk kefir is a fermented drink obtained by the fermentation of milk with kefir grains, a symbiotic community of bacteria and yeast confined in an exopolysaccharide matrix (1). *Lactobacillus kefiranofaciens* has been described as the dominant species of kefir grains and the main producer of the grain matrix (2, 3). *Lactobacillus helveticus* is an important inhabitant of kefir with reported production of antimicrobial peptides (4). *L. helveticus* OSU-BDGOAK2 and *L. kefiranofaciens* OSU-BDGOA1 were previously isolated from kefir grains and identified by amplification of the 16S rRNA gene, both strains exhibited *in vitro* antibacterial activity (5).

Glycerol stocks were activated by plating on Man Rogosa-Sharp (MRS; Difco, Franklin Lakes, NJ, USA) agar and incubated anaerobically at 37°C for 48 h. Genomic DNA extraction was performed using the PowerSoil Pro (Qiagen, Redwood City, CA,USA) kit automated for high throughput on the QiaCube HT (Qiagen), using PowerBead Pro Plates (Qiagen) with 0.5 and 0.1 mm ceramic beads. DNA quantification was performed with Quant-iT PicoGreen dsDNA Assay (Invitrogen, USA). DNA library creation and sequencing was performed by Diversigen Inc. (Minneapolis, MN, USA). DNA was prepared for sequencing with a procedure adapted from the Nextera Library Preparation Kit (Illumina, USA). Libraries were then sequenced on an Illumina NovaSeq 6000 (v1.5) with an S1 flowcell using paired-end 2 × 150 bp reads (targeting 2M reads/sample), the quality of the reads was assessed by filtering for low quality (Q-Score < 30) and length (<50), and paired-adapter sequences were trimmed using cutadapt (6). The resulting reads were assembled into contigs using SPAdes (v3.11.0) (7). Contigs greater than 1,000 bases in length were used in a QUAST (v4.5) (8) analysis. Genome annotation of predicted genes was performed using RAST (v2.0) (9) and the NCBI Prokaryotic Genome Annotation Pipeline (PGAP, v6.5). Prophage Hunter was used to identify potential prophages (10). Default parameters were used for all software unless otherwise specified.

The annotation showed a total of 2,077 genes for OSU-BDGOAK2 and 2,084 for OSU-BDGOA1, respectively (Table 1). The genomes were further analyzed with BAGEL4 (11) for the detection of bacteriocins. Three genes for Helveticin-J, one Enterolysin A, and a post-translationally modified peptide LAP (Linear azol(in)e-containing peptide) were found by BAGEL4 in OSU-BDGOAK2, while Helveticin-J and Enterolysin A genes were found in OSU-BDGOA1. Gene clusters involved in Type III PKS-derived polyketides synthesis were found in both genomes (12), while OSU-BDGOA1 harbors genes predicted to be involved in exopolysaccharide production, similar to the ones found in *L. kefiranofaciens* ZW3 genome (13). Lactobacillus phage LfeInf (74% identity) sequence was found in OSU-BDGOA1 and phage phiAQ113 (82% identity) was identified in OSU-BDGOAK.

**TABLE 1:** Characteristics of bacterial isolates

Strain	OSU-BDGOAK2	OSU-BDGOA1
GenBank no.	JARXPA000000000	JARJVW000000000
Genome size (bp) *^a^*	1,929,111	2,063,076
Coverage (×)	599.47	741.14
GC content (%)	36.6	37.9
No. of contigs	118	64
Contig N_50_ (bp)	25,773	71,931
No. of predicted genes	2,077	2,084

^a^ bp, base pairs.

## Data Availability

The draft genomes were deposited in the NCBI under the Bioproject PRJNA892155 with SRA accession numbers SRR21977427 and SRR21986276 and BioSamples SAMN31370629 and SAMN31370630.
